# A rare presentation of small bowel obstruction

**DOI:** 10.1259/bjrcr.20150310

**Published:** 2016-07-08

**Authors:** Sharif Darwish, Daniel J Bell

**Affiliations:** Department of Radiology, North Middlesex University Hospital NHS Trust, London, UK

## Abstract

A 92-year-old, 41 kg female presented with a 4-day history of abdominal distension, intermittent lower abdominal pain, nausea, vomiting and lack of bowel opening. Contrast-enhanced CT scan of the abdomen and pelvis showed multiple dilated small bowel loops, secondary to incarceration of the ileum in an obturator hernia (OH) on the right. The patient underwent an emergency laparotomy with reduction of the OH. The small bowel was viable and no resection was required. OH is a rare but significant cause of small bowel obstruction, with a high mortality rate. Of all the imaging modalities reviewed, CT scan is highly effective in reducing diagnostic delay, and ultimately can reduce morbidity and mortality rates of patients presenting with an incarcerated OH.

## Clinical presentation

A 92-year-old, 41 kg female presented with a 4-day history of abdominal distension, intermittent lower abdominal pain, nausea, vomiting and lack of bowel opening. Her past medical history was remarkable for a low-grade small bowel obstruction (SBO) 3 years previously, which had been treated conservatively. On examination, she appeared dehydrated, with a soft tender abdomen and increased frequency of bowel sounds. There were no palpable hernias. Rectal examination was unremarkable.

## Differential diagnosis

SBO, unknown cause. Adhesions were unlikely in this virgin abdomen.

## Investigations/imaging findings

Laboratory markers were unremarkable, including normal white cell count, C-reactive protein, serum lactate and amylase levels.

### Abdominal plain film

Multiple loops of dilated small bowel; large bowel not clearly seen, suspicious for distal SBO. Calcified fibroids in the pelvis. No free gas.

### CT abdomen/pelvis + contrast

**Figure 1.**
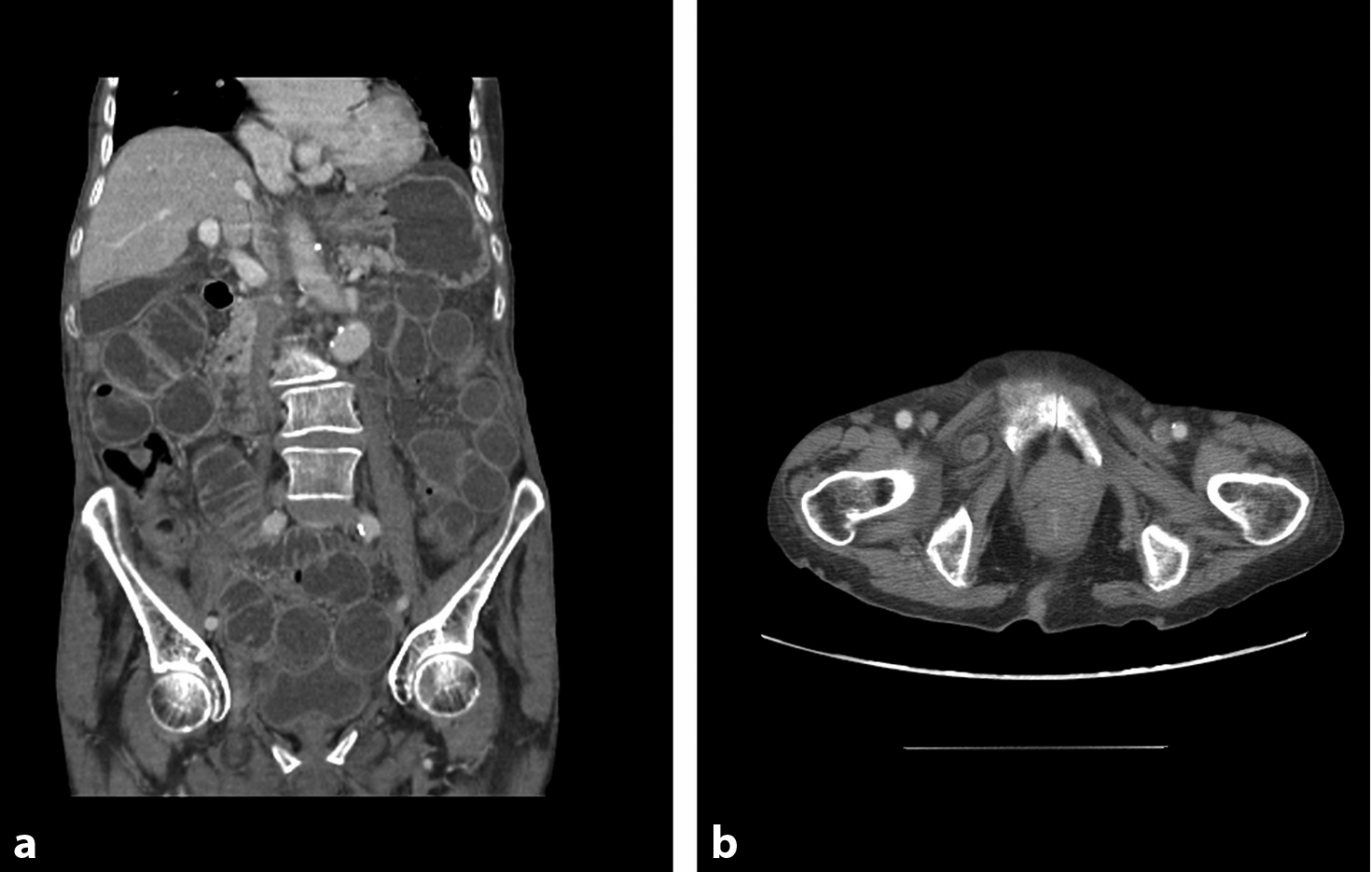
(a) Coronal CT scan of the abdomen and pelvis showing incarceration of a portion of the ileum in a right obturator hernia with transition point. (b) Axial CT scan of the pelvis showing a portion of the ileum in a right obturator hernia.

Multiple dilated small bowel loops secondary to incarceration of the ileum in an obturator hernia (OH) on the right. No evidence of perforation or bowel ischaemia (Figure 1a,b).

### Impression

Distal SBO secondary to incarceration of small bowel in a right OH.

## Treatment

After review by the surgical team and owing to concern of bowel ischaemia, the patient had an emergency laparotomy with reduction of the OH. A Richter’s type OH was found with the mid-ileum incarcerated within the right obturator foramen. The hernia was reduced and the hernia sac plicated; the small bowel was viable and no resection was required.

## Outcome and follow-up

After an uneventful recovery, the patient was discharged home.

## Discussion

An OH is a rare hernia with an incidence of 0.073% of all hernias.^1^ The rate of OH causing SBO that requires surgical management is 0.02%.^1^ Although rare, incarcerated OH can have a mortality rate as high as 70%, and it is for this reason that an accurate and fast diagnosis is made to guide surgical management.^2^ In 90% of cases, the most common clinical presentation is intestinal obstruction.^2^ Although not present in this case, OH is associated with other classical signs such as the Howship–Romberg sign and the Hannington-Kiff sign.^3^ OH is more common in females than males (ratio 6 : 1) and classically presents in elderly, emaciated multiparous females.^2^ It is for this reason that it has been named the “skinny old lady hernia. Risk factors are associated with conditions that increase intra-abdominal pressure and include chronic constipation, multiparity, chronic obstructive pulmonary disease, ascites and kyphoscoliosis.^2^

There are three stages of formation of an OH. The first (prehernial) stage is characterized by preperitoneal fatty tissue entering the pelvic orifice of the obturator canal. The second (developmental) stage begins when minimal changes progress to true sac formation. The third stage starts with the onset of clinically significant symptoms.^3^

There are three different anatomical variations in the pathway facilitating OH formation.^3^ The first and most common is *via* the external orifice of the obturator canal.^3^ The sac is found anterior to the external obturator muscle and inferior to the pectineus muscle. This is *via* the same route that the anterior division of the obturator nerve follows.^3^ Our case represents the first and most common variation. The second variation follows the route of the posterior division of the obturator nerve, and the hernia lies between the middle and superior fascicles of the obturator externus muscle.^3^ The sac is posterior to the adductor brevis muscle. The third is the rarest variation, with the sac found between the internal and external obturator membranes and muscles.^3^

A variety of modalities can be used to image OH: abdominal radiography (AXR), ultrasound, CT and MRI. Diagnosing SBO pre-operatively using clinical findings, laboratory tests, AXR and single-detector CT scan has been found in historical series to be reliable in only 15–50% of patients.^4^ The appearance of an incarcerated hernia on AXR shows gas in the area of the obturator foramen with small bowel loop dilatation.^5^ Although cost-effective, an AXR is diagnostic in only 50–60% of cases;^4^ indeed in 21% cases, the AXR was apparently completely unremarkable.^4^

Ultrasound was able to successfully diagnose bowel obstruction secondary to OH in a small case series of four patients.^6^ Sonographic signs suggestive of incarceration in all forms of SBO secondary to hernia are free fluid in the hernia sac, fluid in the herniated bowel loop, bowel wall thickening in the hernia and dilated bowel loops in the abdomen.^7^ These signs are found in a high percentage of cases and one study reported that ultrasound was highly accurate in diagnosing incarcerated hernias, with a false-positive result of only 2 out of 126 non-incarcerated hernias.^7^ However, this study included only one incarcerated OH.

Although its use is uncommon in the acute setting, ultrasound imaging combines the benefits of being radiation-free and able to be used at the bedside in an emergency setting. In expert hands, ultrasound examination through the flanks can evade bowel gas and correctly identify the level of SBO.^4^ However, there are limitations as it may not detect obstructions in deep abdominal loops, especially in patients with poor acoustic windows.^6^ Ultrasound is less sensitive at detecting low-grade SBOs and is rarely used in the acute setting of SBO. This is owing to reduced sensitivity and high variability in operator technique and ability.

Equivocal or negative findings on AXR or ultrasound imaging in a patient presenting with symptoms suggestive of obstruction require further imaging. CT scan with intravenous contrast medium is considered the initial imaging of choice for SBO in suspected patients with a very high sensitivity for high-grade obstruction. Not only can CT be used to assess the site and severity of SBO, it is also particularly useful in identifying closed-loop obstructions and strangulation.^4^ The identification of bowel ischaemia has been aided by the use of faster multidetector CT scanners with multiplanar reformatting to identify arterial and venous bowel wall enhancement,^4^ both of which can alter the decision for rapid surgical intervention.

Fluoroscopic contrast studies have also been largely superseded by CT scan. This is owing to the disadvantages of the patient having to swallow large amounts of contrast agent, inferior diagnostic accuracy and the time taken to perform this test in the setting of an acute abdomen.^4^

However, combining CT scan with oral contrast affords a superior visualization of the small bowel.^4^ Neutral oral contrast allows for accurate determination of bowel wall thickness and assessment of mucosal hyperaemia.^4^ Positive oral contrast is useful for visualizing the presence of adhesions and fistulae, and is ideal in cases in which intravenous contrast is contraindicated.^4^ The use of oral contrast can result in the appearance of pseudomasses or pseudofold thickening, which has the potential for introducing error in image interpretation.^4^ These errors can be reduced with the use of intravenous contrast.

CT enteroclysis combines the advantages of fluoroscopy and conventional CT in a clinically obstructed patient, overcoming the difficulties encountered with each individual technique.^4^ CT enteroclysis requires the infusion of a contrast agent *via* long nasointestinal tubes and distension of the bowel wall, which overcomes the shortfalls of abdominal CT scan in diagnosing low-grade SBO. CT enteroclysis is seldom used to evaluate acute SBO, not least because of its invasiveness and difficulty, and is a technique usually reserved for tertiary centres.

MRI is more favourable for imaging low-grade SBO, particularly MR enteroclysis. This is because it can be used to monitor small bowel filling in real time, reducing the risk of exposure to ionizing radiation, thus making it superior to CT enteroclysis in assessing low-grade SBO.^8^ It is for this same reason that MRI would be favoured in assessing high-grade SBO in pregnant females or children. MR enteroclysis also improves the distension of small bowel, allowing for the detection of subtle transition points in the more distal small bowel, which can be missed with other techniques.^8^ Compared with CT enteroclysis, MR enteroclysis offers the advantages of direct multiplanar imaging, soft-tissue contrast and real-time acquisition of function information without the risk of ionizing radiation.^4^ However, MRI requires much longer acquisition times than CT, is less widely available for acute patients and is therefore rarely used in an emergency setting.

A study of 36 cases of OH comparing the rates of accurate pre-operative diagnoses before and after the use of CT demonstrated an improvement from 39% to 78%.^9^ However, the authors found that, despite this, the use of CT did not improve post-operative complications or overall mortality. This finding could be compounded by many factors, including delay in patient presentation from onset of symptoms, time from presentation to imaging and also patient comorbidities.^9^ Similarly, a retrospective case series over 58 years, analyzing 30 patients who underwent OH repair demonstrated that the use of CT reduced the likelihood of developing a post-operative complication of any type, odds ratio 0.08 (*p* = 0.04).^10^ However, this study did not show any improvement in mortality with pre-operative CT. This finding could be inaccurate for a number of reasons, such as small sample size and timing of CT scan with respect to admission and presentation of symptoms.^10^ Even if this finding was found to be significant, it does not account for other benefits of pre-operative CT scan, such as allowing the surgeon to plan the surgical approach.^10^

Conversely, a study of 43 cases of patients with OH undergoing surgery showed that the use of CT scanning pre-operatively resulted in a statistically significant lower rate of gut resection and mortality.^11^ It also showed that CT scanning significantly increased the diagnostic accuracy in patients with OH.^11^ In a case series of 16 patients with incarcerated OH requiring surgery, 3 patients were imaged using CT and all were correctly diagnosed pre-operatively. The remainder of the cases were diagnosed intraoperatively. In a further study, CT scan was performed to confirm the diagnosis of OH in eight patients with 100% accuracy.^12^

## Learning points

1. OH is a rare but significant cause of SBO with a high mortality rate.
2. There are three states of formation of an OH, with also three different anatomical variations in its presentation.
3. Contrast-enhanced CT is highly effective in reducing diagnostic delay and can ultimately reduce morbidity and mortality rates of patients presenting with an incarcerated OH.

## Consent

The patient of our case report is unfortunately deceased. We have been unable to obtain informed consent from the next of kin despite exhaustive attempts to contact them. The paper has been sufficiently anonymized so as not to cause harm to the patient or her family.
